# Implementation tendencies and expert perspectives on physician-performed prehospital endotracheal intubation in Japan: Findings from the first round of a Delphi survey

**DOI:** 10.1371/journal.pone.0346146

**Published:** 2026-03-30

**Authors:** Hiroaki Taniguchi, Kazuhiko Omori, Hiroki Nagasawa, Hiromichi Ohsaka, Youichi Yanagawa

**Affiliations:** 1 Emergency and Disaster Medicine, Juntendo University Graduate School of Medicine, Tokyo, Japan; 2 Acute Critical Care Medicine, Shizuoka Hospital, Juntendo University, Shizuoka, Japan; Jazan University College of Applied Medical Science, SAUDI ARABIA

## Abstract

Standardization of prehospital endotracheal intubation (ETI) has been shown to be essential for improving first-pass success rates and minimizing complications. In Japan, however, physician-performed prehospital ETI lacks uniform implementation. To address this gap, we conducted a Delphi study to assess expert consensus on key practices and discrepancies between perceived appropriateness and actual use. We conducted the first round of a modified two-round Delphi study using a web-based questionnaire comprising 90 recommendation statements across eight domains, derived from the literature and a pilot study. Eligible participants were physicians with at least three years of prehospital emergency care experience and a minimum of 30 prehospital ETI cases. Statements were rated for perceived appropriateness and implementation on a 4-point Likert scale, with consensus defined as a positive response rate of ≥70.0%. Free-text comments were also collected to explore areas of discrepancy. Among 58 invited physicians, 40 (69.0%) responded, of whom 65.0% had more than 15 years of prehospital experience. Consensus on appropriateness was achieved for 72 of 90 items (80.0%), whereas consensus on implementation was reached for 57 items (63.3%). At the domain level, appropriateness consensus was highest in the Confirmation domain (100%; 9/9 items) and lowest in the Environment during endotracheal intubation domain (25%; 1/4 items). Discrepancies between perceived appropriateness and implementation were observed across multiple domains, including peripheral equipment and medication-related practices. At the item level, checklist use was considered appropriate by 65.0% of respondents but reported as implemented by only 10.0%. These findings highlight gaps between perceived appropriateness and implementation of physician-performed prehospital ETI practices in Japan, underscoring the need for context-sensitive, locally adapted protocols.

## Introduction

Prehospital endotracheal intubation (ETI) is a vital procedure for patients in life-threatening conditions, with first-pass success rates for physician-performed ETI reported between 86.2% and 96.6% [1–3]. Given the high risk of complications such as hypoxia, hypotension, and aspiration, optimizing success is essential [4]. ETI is a complex process that requires appropriate patient assessment, medication selection, environmental control, and communication among team members. Previous studies have shown that not only procedural techniques but also provider experience scene environment, and the use of checklists and protocols contribute to improving first-pass success and patient outcomes [2,5–10].

Standardized protocols for prehospital ETI and anesthesia have been developed and evaluated in regions such as North America, Europe, and Australia. These protocols typically cover preparation, induction, intubation, and post-intubation management, and have been associated with improved first-pass success rates and a potential reduction in early mortality [2,9–11] They also improve educational outcomes and facilitate continuous quality improvement through the collection of uniform data [12,13].

In Japan, physician-staffed prehospital care via rapid response cars and helicopter emergency medical services (HEMS) has expanded. However, several challenges hinder the standardization of physician-performed prehospital ETI. Practice settings, provider training, and available resources vary across regions, and no national guidelines currently exist [14]. As a result, outcome data related to physician-performed prehospital ETI remain limited and heterogeneous, making systematic evaluation and generalization within the Japanese context challenging.

This study aimed to explore expert perspectives on standardizing physician-performed prehospital ETI in Japan. Given the lack of formal domestic guidelines and operational variability, we employed a structured consensus process using the Delphi method [15]. Here, we present the first-round findings, comparing perceived appropriateness and implementation tendencies with internationally reported practices.

## Materials and methods

### Study design and setting

This study was conducted in accordance with the ACcurate COnsensus Reporting Document (ACCORD) guideline [15,16], which provides comprehensive reporting standards for consensus methods in health-related research. We analyzed the first round of a planned two-round modified Delphi process. In this modified Delphi design, predefined items derived from the literature review and a pilot study were presented from the first round, reflecting a confirmatory consensus approach rather than open item generation [17]. A completed ACCORD checklist with page references is provided in S1 Table. The protocol for this prospective study was approved by the institutional review board of our university (Approval Number: E24-0449-S01). All procedures adhered to the principles of Good Clinical Practice and the Declaration of Helsinki.

### Identification of Delphi items

Initial candidate items for the Delphi process were identified through a combination of literature review and a pilot study. S1 Fig. outlines the overall study process.

For the literature review, PubMed/MEDLINE was searched in November 2024 to identify studies published between 2015 and 2024. Eligible studies addressed prehospital care in combination with ETI or anesthesia. As the objective of the review was to inform item generation for the Delphi process, studies relevant to the standardization of prehospital ETI were considered, and heterogeneity in study design, target populations, and outcome measures was accepted. Studies were excluded if they (1) were conducted exclusively in emergency department settings, (2) were limited to intensive care unit settings, or (3) involved paramedic-performed ETI. Articles were screened by title and abstract by one reviewer (H.T.) and assessed for eligibility by a second author (K.O.). A total of 177 studies were included for item generation. From these sources, 79 preliminary items were extracted for the Delphi process.

**Fig 1:**
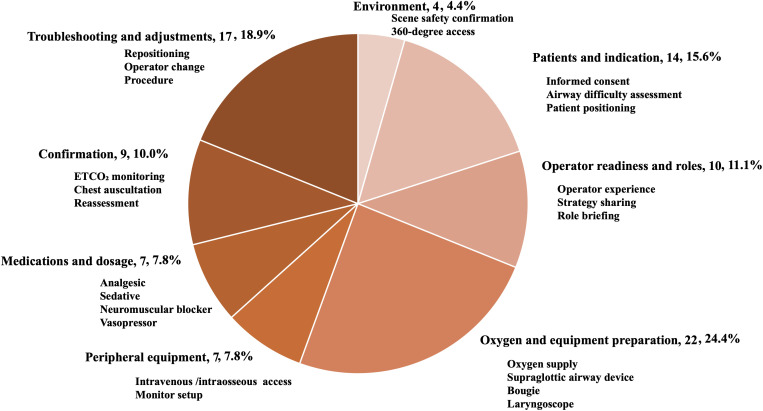
Distribution of recommendation items across eight domains.

These items were evaluated in a pilot study conducted in December 2024 by three authors (H.N., H.O., and Y.Y.), who independently reviewed the draft items and provided feedback on content appropriateness and completeness. Based on their input, a supplementary manual search was conducted to include additional topics suggested during the pilot study. Several additional practices were added, including laryngoscope selection according to patient characteristics, assessment of dental stability, equipment preparation such as stethoscope availability, and confirmation procedures after ETI. The final list comprised 90 items. These items were categorized by the authors along a chronological axis—ranging from initial preparation to induction, confirmation, and troubleshooting—and grouped into eight domains (Fig 1).

Derived from the literature and a pilot study, a total of 90 recommendation items were organized along a chronological axis from preparation to troubleshooting and grouped into eight procedural domains. The number and percentage of items in each domain are indicated, illustrating the relative emphasis across phases of prehospital endotracheal intubation.

### Selection and description of participants

The Delphi process was coordinated by K.O., a board-certified specialist in emergency medicine, trauma, critical care, and aeromedical services in Japan. While no universally accepted guideline exists for expert panel selection [18,19], we considered the specific characteristics of Japan’s prehospital care system. Physicians with at least 3 years of experience in doctor car or HEMS operations and a minimum of 30 prehospital ETI cases were eligible. No patient or public involvement was included in any step of the study. A target sample size of 40 was set. Invitations and communications were managed centrally by K.O., conducted either in person or online.

### Consensus process, data collection, and measurements

Participants completed the survey individually via Google Forms (Googleplex, California, USA). The coordinator also participated in voting, as permitted by the study design. At the beginning of the survey, participants were provided with information on the study’s purpose, potential benefits and risks, and confidentiality, and electronic informed consent was obtained before proceeding. Participants were first asked to indicate their demographic and professional characteristics, and then to rate the appropriateness and implementation status of each item using the 4-point Likert scale (1 = Not applicable, 2 = Somewhat not applicable, 3 = Somewhat applicable, 4 = Highly applicable). While justifications were not required, participants were encouraged to provide optional free-text comments or suggest additional items.

Although definitions of consensus vary across studies, thresholds around 70–80%, with a median of 75%, are commonly used [20,21]. In this study, consensus was defined as ≥70% agreement (ratings of 3 or 4 on the Likert scale), applied as a pragmatic screening threshold in this first-round exploratory analysis to allow broad item inclusion for subsequent refinement. Free-text responses were coded and reviewed by two authors (H.T. and K.O.) to identify emerging themes. All data were stored electronically in a secure, password-protected folder. To reduce respondent burden and improve accessibility, the survey was conducted in Japanese, with multiple reminders and a modest honorarium provided to encourage participation. No feedback was provided, as this report covers the first round only.

### Statistics

Responses to each of the 90 recommendation items were summarized using descriptive statistics. Item-level agreement rates for perceived appropriateness and implementation were calculated, and the number of items reaching the predefined consensus threshold was summarized. Domain-level distributions were summarized using medians and interquartile ranges (IQRs). Discrepancies between perceived appropriateness and implementation were examined across and within the eight domains. Free-text responses were narratively reviewed to identify contextual factors potentially influencing observed variation.

## Results

### Panel characteristics

The survey was conducted from February 24 to March 17, 2025. Of the 58 physicians invited, 40 (69.0%) responded. No protocol deviations occurred during the first round of the Delphi process. Panel characteristics are shown in Table 1. All participants were male emergency physicians, with only 2.5% also board-certified in anesthesiology. Sixty-five percent had more than 15 years of prehospital care experience. Among their affiliated institutions, 27.5% reported access to hands-on training, while 37.5% reported no formal training.

**Table 1:** Characteristics of participants in the first-round Delphi survey.

Variable	n (%)
Age group	
< 40 years	3 (7.5)
40–49 years	15 (37.5)
50–59 years	15 (37.5)
≥ 60 years	7 (17.5)
Gender, male	40 (100)
Years of clinical experience	
< 5 years	0 (0)
5–9 years	2 (5.0)
10–14 years	3 (7.5)
15–19 years	6 (15.0)
≥ 20 years	29 (72.5)
Years of experience in prehospital emergency medicine	
< 5 years	1 (2.5)
5–9 years	5 (12.5)
10–14 years	8 (20.0)
15–19 years	12 (30.0)
≥ 20 years	14 (35.0)
Board certifications (multiple responses allowed)	
Certified in Emergency Medicine	40 (100)
Certified in Anesthesiology	1 (2.5)
Certified in General Surgery	8 (20.0)
Certified in Intensive Care Medicine	19 (47.5)
Certified in Trauma Surgery	16 (40.0)
Certified in Aeromedical Medicine	34 (85.0)
Other*	6 (15.0)
Number of prehospital endotracheal intubation	
30–50 cases	9 (22.5)
51–100 cases	11 (27.5)
101–150 cases	4 (10.0)
151–200 cases	2 (5.0)
> 201 cases	14 (35.0)
Annual number of dispatches at participants’ facilities	
< 301 cases	6 (15.0)
301–500 cases	13 (32.5)
501–700 cases	3 (7.5)
701–1000 cases	5 (12.5)
> 1001 cases	13 (32.5)
Presence of formal intubation training at participants’ facility	
Clinical training (e.g., anesthesiology rotation)	11 (27.5)
Simulation-based training	12 (30.0)
Didactic lectures	2 (5.0)
No formal training	15 (37.5)

*Other includes Internal Medicine (n = 1), Orthopedic Surgery (n = 3), and Neurosurgery (n = 2).

### Consensus rates and domain-level trends

Table 2 summarizes domain-level consensus and agreement rates, while S2 Table presents the full distribution of item-level responses across the 4-point Likert scale for both perceived appropriateness and implementation. Of the 90 items, 72 (80.0%) reached the predefined consensus threshold for appropriateness, while 57 (63.3%) reached consensus for implementation. At the domain level, the highest proportion of items reaching the consensus threshold for appropriateness was observed in the Confirmation domain (100%; 9/9 items), whereas the lowest was observed in the Environment during endotracheal intubation domain (25%; 1/4 items).

**Table 2:** Domain-level summary of consensus and agreement rates.

Domain	Items, n	Appropriateness		Implementation	
		Consensus* n (%)	Agreement Rate† Median (IQR, %)	Consensus* n (%)	Agreement Rate† Median (IQR, %)
Environment during endotracheal intubation	4	1 (25.0)	66.3 (59.3-91.9)	1 (25.0)	51.3 (16.3-82.5)
Patients and indication	14	12 (85.7)	88.8 (81.3-95.0)	10 (71.4)	81.3 (59.4-92.5)
Operator readiness and role assignments	10	6 (60.0)	83.8 (59.4-93.1)	5 (50.0)	65.0 (25.0-80.0)
Oxygen and equipment preparation	22	21 (95.5)	95.0 (86.9-98.1)	19 (86.4)	88.8 (78.8-95.0)
Peripheral equipment	7	6 (85.7)	82.5 (75.0-100)	3 (42.9)	67.5 (42.5-97.5)
Medications and dosage considerations	7	5 (71.4)	82.5 (60.0-87.5)	3 (42.9)	67.5 (30.0-80.0)
Confirmation	9	9 (100.0)	97.5 (90.0-97.5)	8 (88.9)	95.0 (76.3-97.5)
Troubleshooting and adjustments	17	12 (70.6)	87.5 (67.5-92.5)	8 (47.1)	65.0 (50.0-78.8)

* Consensus indicates the number (%) of items reaching the predefined threshold (≥70.0%) for agreement (ratings of 3 or 4 on the 4-point Likert scale).

† Agreement rate indicates the median (IQR, %) of item-level agreement rates, defined as the percentage of respondents rating an item as 3 or 4.

At the domain level, agreement rates further characterized the alignment between perceived appropriateness and actual implementation. Across all domains, agreement rates for implementation were lower than those for appropriateness. Such differences were observed in domains characterized by high agreement on appropriateness but lower agreement on implementation, including peripheral equipment and medications and dosage considerations (median appropriateness agreement, 82.5% for both; median implementation agreement, 67.5% for both). Similar patterns were also observed in domains involving role assignments, troubleshooting, and environmental considerations.

These discrepancies were also evident at the item level. For example, checklist use was rated as appropriate by 65.0% of respondents, whereas only 10.0% reported that it was routinely implemented.

### Contextual insights from free-text comments

S1 File summarizes the free-text responses. Comments highlighted contextual differences, including the typical use of ambulances in Japan for prehospital ETI, limited availability of certain medications (e.g., succinylcholine, ketamine), and restricted access to devices such as supraglottic airway tools, bougies, and ultrasound. Respondents also expressed differing views on laryngoscope preference (video vs. direct), drug protocols, and team structure.

## Discussions

This study examined expert opinions on physician-performed prehospital ETI in Japan via a first-round consensus process. While most items were considered appropriate, only 63.3% were reportedly implemented. Substantial gaps were observed between appropriateness and implementation, particularly regarding checklists, peripheral equipment, and medication protocols.

International efforts to standardize prehospital ETI have advanced in North America, Europe, and Australia [2,8,10,22,23]. These typically address key domains such as environment, assessment, airway preparation, drug administration, and troubleshooting [7,24,25]. In contrast, Japan lacks national guidelines, resulting in fragmented training and practice across institutions. In our study, only 27.5% of participants reported access to hands-on training, and 37.5% reported no formal training, suggesting a potential need for nationwide education frameworks. For example, although 65.0% rated checklist use as appropriate, only 10.0% reported its routine implementation.

This study revealed clear variation across domains in the alignment between perceived appropriateness and actual implementation. Domains such as Patients and Indication, Oxygen and Equipment Preparation, and Confirmation showed consistently high ratings for both appropriateness and implementation, suggesting that these practices are widely accepted and feasible within routine prehospital workflows. These domains likely represent foundational elements of airway management that require minimal additional resources. In particular, the uniformly high agreement observed in the Confirmation domain may reflect the recognition of these items as fundamental safety practices, although such consensus could also be influenced by shared normative expectations among clinicians.

In contrast, lower implementation levels were observed in domains such as Medication administration, Peripheral Equipment, and Role Assignments, despite moderate to high perceived appropriateness. These domains appear more vulnerable to contextual constraints related to training, staffing structure, and resource availability. To better understand these gaps, the observed discrepancies can be broadly interpreted through three interrelated dimensions: educational, structural, and operational factors.

From an educational perspective, substantial heterogeneity was observed in provider experience, training opportunities, and procedural preparedness among prehospital physicians. In Japan, prehospital care is predominantly delivered by physicians with emergency medicine backgrounds, and access to structured airway training varies across institutions. This variability may particularly affect the implementation of practices requiring advanced pharmacologic management or adjunct airway devices. Similar education-related challenges have been reported in other physician-staffed prehospital systems operating under staffing and resource constraint [26–28].

Structural and operational characteristics of Japanese prehospital care may further limit the feasibility of implementing certain airway management practices. Free-text comments indicated that ETI is frequently performed by a single physician, often without designated assistants or clearly defined role assignments, constraining task delegation and team-based approaches. Respondents also described variability in equipment availability, medication stocking, and procedural environments across institutions. Environmental constraints, including limited procedural space and difficulty interpreting environment-dependent concepts such as “360-degree access” in ambulance-based settings, further illustrate challenges in directly applying internationally recommended practices to the Japanese prehospital context.

This study represents the first nationwide and structured effort to explore consensus among experts on physician-performed prehospital ETI in Japan. By evaluating both the perceived appropriateness and implementation status of internationally derived recommendations, the study revealed discrepancies between conceptual agreement and actual clinical practice, as well as contextual and institutional differences. These findings provide valuable groundwork to inform the development of practical and context-specific protocols. Further consensus-building may help refine expert perspectives and inform feasible, evidence-informed guidelines for the Japanese setting. Beyond consensus efforts, future research should focus on evaluating real-world protocol implementation and the role of structured education and simulation-based training within physician-staffed prehospital systems.

Several limitations should be acknowledged. First, the participant pool was demographically and professionally unbalanced, consisting exclusively of male physicians, most of whom were board-certified in emergency medicine, with relatively limited participation from anesthesiologists. This may have introduced a degree of bias in the perspectives captured, and broader representation would strengthen future rounds of the consensus process. In addition, detailed information on institutional characteristics was not collected, which may have influenced reported practice patterns. Second, the study reflects only the first round of a Delphi-style process, and further rounds are needed to achieve more robust consensus. Some variability in item interpretation was observed, which is inherent to early-round Delphi processes, and the influence of subjective judgments cannot be fully excluded. Third, the recommendation items were generated through a broad, structured review of international literature, which necessarily encompassed heterogeneous evidence quality, clinical contexts, and implementation levels. Accordingly, the content validity of individual items should be regarded as provisional and exploratory at this stage. In addition, the use of a relatively modest consensus threshold (≥70.0%) was intended for first-round screening rather than definitive endorsement, with item refinement and validation anticipated in subsequent Delphi rounds.

## Conclusions

This study identified key discrepancies between expert agreement and reported implementation regarding physician-performed prehospital ETI practices in Japan. The findings offer an important foundation for future standardization efforts tailored to the specific characteristics of Japanese prehospital care.

## Supporting information

S1 Table ACCORD Checklist: Recommended Reporting Items for Studies Employing Consensus Methodologies. Checklist used to ensure transparent reporting of the modified Delphi process, including page references. The ACCORD (ACcurate COnsensus Reporting Document) checklist is intended to support transparent reporting of studies using consensus methods. It is best used alongside the Explanation and Elaboration article, available via the ACCORD website (https://www.ismpp.org/accord) and the EQUATOR Network.
(DOCX)

S2 Table Full response distributions for appropriateness and implementation ratings of recommendation items. This table presents the complete distribution of responses across the 4-point Likert scale (ratings 1–4) for each recommendation item, allowing detailed inspection beyond binary consensus classification.
(DOCX)

S1 Fig Identification and Development Process of Delphi Survey Items. Flowchart illustrating the process of item generation for the Delphi survey, including literature review, pilot testing, and final categorization. Candidate items were identified through a literature review and a pilot study, followed by supplementary manual searches based on pilot feedback. The final set of items was organized chronologically and grouped into eight domains.
(DOCX)

S1 File Summary of free-text comments from round 1. Thematic analysis of contextual factors and suggestions raised by participants in open comment fields.
(DOCX)
