# Supplementary material for: Implementation tendencies and expert perspectives on physician-performed prehospital endotracheal intubation in Japan: Findings from the first round of a Delphi survey
Source: PLoS One. 2026 Mar 30;21(3):e0346146. doi: 10.1371/journal.pone.0346146 (PMC13035153; doi:10.1371/journal.pone.0346146)
Supplement: S2 Table — This table presents the complete distribution of responses across the 4-point Likert scale (ratings 1–4) for each recommendation item, allowing detailed inspection beyond binary consensus classification. (DOCX) [file pone.0346146.s002.docx]

**Table S2. Full response distributions for appropriateness and implementation ratings of recommendation items**

| Domain | Recommended item | Appropriateness (Likert 1–4), n (%) | | | | Implementation (Likert 1–4), n (%) | | | |
| --- | --- | --- | --- | --- | --- | --- | --- | --- | --- |
|  |  | 1 | 2 | 3 | 4 | 1 | 2 | 3 | 4 |
| Environment during endotracheal intubation | |  |  |  |  |  |  |  |  |
|  | Share the checklist | 5 (13%) | 9 (23%) | 20 (50%) | 6 (15%) | 29 (73%) | 7 (18%) | 2 (5%) | 2 (5%) |
|  | Check that the scene and surrounding area are safe and under appropriate control | 0 (0%) | 0 (0%) | 11 (28%) | 29 (73%) | 2 (5%) | 3 (8%) | 13 (33%) | 22 (55%) |
|  | Move to the ambulance or consider moving | 3 (8%) | 10 (25%) | 12 (30%) | 15 (38%) | 2 (5%) | 11 (28%) | 16 (40%) | 11 (28%) |
|  | Have access to the patient from all 360 degrees | 5 (13%) | 12 (30%) | 17 (43%) | 6 (15%) | 8 (20%) | 18 (45%) | 12 (30%) | 2 (5%) |
| Patients and indication | |  |  |  |  |  |  |  |  |
|  | Explain to the patient and obtain informed consent | 1 (3%) | 5 (13%) | 16 (40%) | 18 (45%) | 2 (5%) | 14 (35%) | 15 (38%) | 9 (23%) |
|  | Assess ventilation difficulties | 0 (0%) | 0 (0%) | 9 (23%) | 31 (78%) | 0 (0%) | 3 (8%) | 14 (35%) | 23 (58%) |
|  | Assess intubation difficulties | 0 (0%) | 2 (5%) | 4 (10%) | 34 (85%) | 0 (0%) | 5 (13%) | 16 (40%) | 19 (48%) |
|  | Consider the use of bag-valve-mask ventilation or supraglottic devices (consider alternatives to intubation) | 1 (3%) | 5 (13%) | 11 (28%) | 23 (58%) | 1 (3%) | 9 (23%) | 14 (35%) | 16 (40%) |
|  | Assess the indications and disadvantages of tracheal intubation | 0 (0%) | 0 (0%) | 8 (20%) | 32 (80%) | 0 (0%) | 0 (0%) | 14 (35%) | 26 (65%) |
|  | If the benefits outweigh the risks in the following conditions, rapid sequence intubation (RSI) should be selected.  Conditions: airway emergency, ventilation failure, impaired consciousness, post-head injury or agitation | 1 (3%) | 2 (5%) | 3 (8%) | 34 (85%) | 1 (3%) | 2 (5%) | 10 (25%) | 27 (68%) |
|  | If the patient does not meet the criteria for rapid sequence intubation (RSI), consider awake or delayed sequence intubation (DSI) | 1 (3%) | 8 (20%) | 14 (35%) | 17 (43%) | 3 (8%) | 9 (23%) | 13 (33%) | 15 (38%) |
|  | Check the patient's loose teeth and dentures | 1 (3%) | 2 (5%) | 4 (10%) | 33 (83%) | 1 (3%) | 3 (8%) | 9 (23%) | 27 (68%) |
|  | Fix the spine if necessary | 0 (0%) | 2 (5%) | 7 (18%) | 31 (78%) | 0 (0%) | 2 (5%) | 14 (35%) | 24 (60%) |
|  | Consider a 15-degree head-up tilt (especially in obese patients and pregnant women) | 1 (3%) | 5 (13%) | 26 (65%) | 8 (20%) | 6 (15%) | 11 (28%) | 20 (50%) | 3 (8%) |
|  | Identify the cricothyroid ligament | 2 (5%) | 13 (33%) | 16 (40%) | 9 (23%) | 12 (30%) | 15 (38%) | 8 (20%) | 5 (13%) |
|  | Perform preoxygenation (15L oxygen administration or back-valve mask) | 2 (5%) | 0 (0%) | 5 (13%) | 33 (83%) | 2 (5%) | 3 (8%) | 9 (23%) | 26 (65%) |
|  | Check that a bougie is advantageous, especially in cases where the airway is contaminated with blood or vomit, the patient is obese, or neck immobilization is required | 2 (5%) | 16 (40%) | 10 (25%) | 12 (30%) | 14 (35%) | 13 (33%) | 7 (18%) | 6 (15%) |
|  | Consider a Macintosh laryngoscope if there is blood, secretions, or vomit in the airway | 1 (3%) | 6 (15%) | 12 (30%) | 21 (53%) | 2 (5%) | 9 (23%) | 13 (33%) | 16 (40%) |
| Operator Readiness and Role Assignments | |  |  |  |  |  |  |  |  |
|  | The person performing the procedure has sufficient experience, is an emergency physician or anesthesiologist who has received training in anesthesia, or has received simulation training | 0 (0%) | 2 (5%) | 14 (35%) | 24 (60%) | 2 (5%) | 6 (15%) | 13 (33%) | 19 (48%) |
|  | The person performing the procedure and the assistants are in the correct positions | 0 (0%) | 1 (3%) | 12 (30%) | 27 (68%) | 0 (0%) | 4 (10%) | 19 (48%) | 17 (43%) |
|  | The person in charge of assisting with intubation is designated and briefed | 0 (0%) | 4 (10%) | 18 (45%) | 18 (45%) | 4 (10%) | 12 (30%) | 15 (38%) | 9 (23%) |
|  | The person in charge of cricoid pressure is designated and briefed | 9 (23%) | 6 (15%) | 20 (50%) | 5 (13%) | 11 (28%) | 19 (48%) | 7 (18%) | 3 (8%) |
|  | The person in charge of time management is designated and briefed | 2 (5%) | 15 (38%) | 13 (33%) | 10 (25%) | 11 (28%) | 18 (45%) | 6 (15%) | 5 (13%) |
|  | The person in charge of administering drugs is designated and briefed | 2 (5%) | 6 (15%) | 13 (33%) | 19 (48%) | 5 (13%) | 7 (18%) | 18 (45%) | 10 (25%) |
|  | The person performing the procedure is designated to take over, or help is available | 8 (20%) | 10 (25%) | 11 (28%) | 11 (28%) | 17 (43%) | 15 (38%) | 5 (13%) | 3 (8%) |
|  | Decide in advance how long you will be involved in the oral tracheal intubation | 4 (10%) | 12 (30%) | 16 (40%) | 8 (20%) | 14 (35%) | 16 (40%) | 7 (18%) | 3 (8%) |
|  | Discuss and share the plan for when the intubation fails | 1 (3%) | 2 (5%) | 14 (35%) | 23 (58%) | 4 (10%) | 6 (15%) | 16 (40%) | 14 (35%) |
|  | Discuss and share the plan for post-intubation management (sedation and respiratory management) | 2 (5%) | 3 (8%) | 12 (30%) | 23 (58%) | 3 (8%) | 5 (13%) | 13 (33%) | 19 (48%) |
| Oxygen and Equipment Preparation | |  |  |  |  |  |  |  |  |
|  | Prepare oxygen supply, check the remaining amount of oxygen cylinders | 0 (0%) | 0 (0%) | 11 (28%) | 29 (73%) | 4 (10%) | 2 (5%) | 13 (33%) | 21 (53%) |
|  | There are spare oxygen cylinders | 1 (3%) | 3 (8%) | 10 (25%) | 26 (65%) | 4 (10%) | 6 (15%) | 9 (23%) | 21 (53%) |
|  | Insert and prepare the airway as necessary | 0 (0%) | 7 (18%) | 10 (25%) | 23 (58%) | 3 (8%) | 8 (20%) | 13 (33%) | 16 (40%) |
|  | The bag valve mask is connected to oxygen and working properly | 0 (0%) | 0 (0%) | 4 (10%) | 36 (90%) | 0 (0%) | 3 (8%) | 5 (13%) | 32 (80%) |
|  | Set up the ventilation circuit | 1 (3%) | 0 (0%) | 10 (25%) | 29 (73%) | 1 (3%) | 4 (10%) | 14 (35%) | 21 (53%) |
|  | You have access to supraglottic devices in an emergency | 4 (10%) | 6 (15%) | 13 (33%) | 17 (43%) | 13 (33%) | 8 (20%) | 8 (20%) | 11 (28%) |
|  | You have access to emergency surgical airway equipment. Prepare a tracheal cannula, scalpel and pean | 0 (0%) | 3 (8%) | 3 (8%) | 34 (85%) | 0 (0%) | 3 (8%) | 11 (28%) | 26 (65%) |
|  | The cuff of the tracheal tube inflates normally and the connector is securely fixed | 0 (0%) | 1 (3%) | 4 (10%) | 35 (88%) | 0 (0%) | 2 (5%) | 8 (20%) | 30 (75%) |
|  | Prepare a syringe for cuff inflation | 0 (0%) | 0 (0%) | 3 (8%) | 37 (93%) | 0 (0%) | 0 (0%) | 3 (8%) | 37 (93%) |
|  | Prepare a (small) tube as a backup | 3 (8%) | 6 (15%) | 13 (33%) | 18 (45%) | 6 (15%) | 11 (28%) | 9 (23%) | 14 (35%) |
|  | The appropriate bougie or stylet is attached or prepared | 1 (3%) | 4 (10%) | 5 (13%) | 30 (75%) | 2 (5%) | 4 (10%) | 9 (23%) | 25 (63%) |
|  | Check the light source for the laryngoscope and the blade of the laryngoscope | 0 (0%) | 0 (0%) | 2 (5%) | 38 (95%) | 0 (0%) | 0 (0%) | 4 (10%) | 36 (90%) |
|  | Prepare a backup blade | 1 (3%) | 4 (10%) | 7 (18%) | 28 (70%) | 2 (5%) | 4 (10%) | 12 (30%) | 22 (55%) |
|  | Prepare a bite block | 0 (0%) | 2 (5%) | 7 (18%) | 31 (78%) | 1 (3%) | 2 (5%) | 7 (18%) | 30 (75%) |
|  | A device for securing the tube is available | 0 (0%) | 0 (0%) | 2 (5%) | 38 (95%) | 0 (0%) | 1 (3%) | 3 (8%) | 36 (90%) |
|  | Select a suction tip device (catheter or Yankauer) | 0 (0%) | 1 (3%) | 5 (13%) | 34 (85%) | 0 (0%) | 2 (5%) | 6 (15%) | 32 (80%) |
|  | Prepare a suction device (on the right side of the patient) | 3 (8%) | 3 (8%) | 8 (20%) | 26 (65%) | 3 (8%) | 5 (13%) | 13 (33%) | 19 (48%) |
|  | Prepare a Magill if foreign objects are a concern | 0 (0%) | 2 (5%) | 5 (13%) | 33 (83%) | 0 (0%) | 3 (8%) | 8 (20%) | 29 (73%) |
|  | Prepare a backup suction device | 5 (13%) | 10 (25%) | 13 (33%) | 12 (30%) | 17 (43%) | 11 (28%) | 8 (20%) | 4 (10%) |
|  | Connect an end-tidal carbon dioxide (EtCO2) monitor | 1 (3%) | 1 (3%) | 5 (13%) | 33 (83%) | 1 (3%) | 3 (8%) | 10 (25%) | 26 (65%) |
|  | Check that the ventilator is working | 1 (3%) | 2 (5%) | 5 (13%) | 32 (80%) | 3 (8%) | 3 (8%) | 3 (8%) | 31 (78%) |
|  | A stethoscope is available | 0 (0%) | 1 (3%) | 2 (5%) | 37 (93%) | 0 (0%) | 1 (3%) | 5 (13%) | 34 (85%) |
| Peripheral equipment | |  |  |  |  |  |  |  |  |
|  | A venous or bone marrow route is secured and reliable | 0 (0%) | 1 (3%) | 6 (15%) | 33 (83%) | 0 (0%) | 2 (5%) | 7 (18%) | 31 (78%) |
|  | The venous route does not interfere with the cuff pressure gauge | 1 (3%) | 6 (15%) | 14 (35%) | 19 (48%) | 1 (3%) | 12 (30%) | 18 (45%) | 9 (23%) |
|  | Monitor the electrocardiogram | 0 (0%) | 0 (0%) | 6 (15%) | 34 (85%) | 0 (0%) | 1 (3%) | 5 (13%) | 34 (85%) |
|  | Monitor the oxygen saturation | 0 (0%) | 0 (0%) | 5 (13%) | 35 (88%) | 0 (0%) | 0 (0%) | 7 (18%) | 33 (83%) |
|  | Set up to measure blood pressure every 2 minutes | 2 (5%) | 6 (15%) | 12 (30%) | 20 (50%) | 3 (8%) | 12 (30%) | 11 (28%) | 14 (35%) |
|  | Install a temperature probe | 5 (13%) | 19 (48%) | 10 (25%) | 6 (15%) | 15 (38%) | 15 (38%) | 5 (13%) | 5 (13%) |
|  | Prepare a gastric tube (especially for children) and be ready to use it if necessary | 3 (8%) | 7 (18%) | 17 (43%) | 13 (33%) | 10 (25%) | 13 (33%) | 9 (23%) | 8 (20%) |
| Medications and Dosage Considerations | |  |  |  |  |  |  |  |  |
|  | Consider premedication with 1-2mg of midazolam or 20-30mg of ketamine (no head trauma) if the patient is agitated | 0 (0%) | 3 (8%) | 14 (35%) | 23 (58%) | 1 (3%) | 7 (18%) | 10 (25%) | 22 (55%) |
|  | Set up a dose of 1-3μg/kg of fentanyl and evaluate side effects | 2 (5%) | 5 (13%) | 13 (33%) | 20 (50%) | 7 (18%) | 6 (15%) | 10 (25%) | 17 (43%) |
|  | Set the dose of ketamine at 1-2mg/kg and evaluate side effects | 6 (15%) | 4 (10%) | 12 (30%) | 18 (45%) | 14 (35%) | 6 (15%) | 9 (23%) | 11 (28%) |
|  | Set the dose of midazolam at 0.01-0.1mg/kg and consider using it in combination with narcotics | 1 (3%) | 5 (13%) | 13 (33%) | 21 (53%) | 3 (8%) | 7 (18%) | 14 (35%) | 16 (40%) |
|  | Set the dose of rocuronium at 1mg/kg | 2 (5%) | 3 (8%) | 9 (23%) | 26 (65%) | 3 (8%) | 5 (13%) | 11 (28%) | 21 (53%) |
|  | If intubation is expected to be difficult, consider using 1.5mg/kg of suxamethonium | 12 (30%) | 5 (13%) | 10 (25%) | 13 (33%) | 20 (50%) | 10 (25%) | 5 (13%) | 5 (13%) |
|  | If hypotension is expected, consider using 0.1mg of phenylephrine | 6 (15%) | 10 (25%) | 14 (35%) | 10 (25%) | 17 (43%) | 11 (28%) | 8 (20%) | 4 (10%) |
| Confirmation | |  |  |  |  |  |  |  |  |
|  | Visually confirm that the tube has passed the vocal cords | 0 (0%) | 1 (3%) | 4 (10%) | 35 (88%) | 0 (0%) | 2 (5%) | 6 (15%) | 32 (80%) |
|  | No obvious air leak | 0 (0%) | 0 (0%) | 4 (10%) | 36 (90%) | 0 (0%) | 1 (3%) | 5 (13%) | 34 (85%) |
|  | Use a qualitative CO2 detector | 7 (18%) | 5 (13%) | 8 (20%) | 20 (50%) | 10 (25%) | 7 (18%) | 6 (15%) | 17 (43%) |
|  | Check chest wall movement | 1 (3%) | 0 (0%) | 1 (3%) | 38 (95%) | 0 (0%) | 0 (0%) | 1 (3%) | 39 (98%) |
|  | Auscultate both axillae and epigastrium | 0 (0%) | 1 (3%) | 4 (10%) | 35 (88%) | 0 (0%) | 1 (3%) | 5 (13%) | 34 (85%) |
|  | Appropriate resistance with bag-valve-mask ventilation | 0 (0%) | 1 (3%) | 7 (18%) | 32 (80%) | 0 (0%) | 2 (5%) | 7 (18%) | 31 (78%) |
|  | Check ventilator parameters | 3 (8%) | 4 (10%) | 7 (18%) | 26 (65%) | 5 (13%) | 3 (8%) | 7 (18%) | 25 (63%) |
|  | Monitor ETCO2 | 1 (3%) | 0 (0%) | 8 (20%) | 31 (78%) | 1 (3%) | 2 (5%) | 11 (28%) | 26 (65%) |
|  | Reassess tube position after each intervention or movement | 0 (0%) | 1 (3%) | 10 (25%) | 29 (73%) | 1 (3%) | 10 (25%) | 11 (28%) | 18 (45%) |
| Troubleshooting and Adjustments | |  |  |  |  |  |  |  |  |
|  | Attempt suction | 1 (3%) | 1 (3%) | 10 (25%) | 28 (70%) | 1 (3%) | 6 (15%) | 11 (28%) | 22 (55%) |
|  | Attempt external laryngeal manipulation | 3 (8%) | 13 (33%) | 15 (38%) | 9 (23%) | 6 (15%) | 17 (43%) | 10 (25%) | 7 (18%) |
|  | Adjust position | 1 (3%) | 4 (10%) | 16 (40%) | 19 (48%) | 2 (5%) | 8 (20%) | 16 (40%) | 14 (35%) |
|  | Try to reposition the bed | 5 (13%) | 8 (20%) | 15 (38%) | 12 (30%) | 8 (20%) | 12 (30%) | 14 (35%) | 6 (15%) |
|  | Adjust the patient's position | 1 (3%) | 4 (10%) | 18 (45%) | 17 (43%) | 2 (5%) | 7 (18%) | 21 (53%) | 10 (25%) |
|  | Insert the blade to the right side of the mouth, avoiding the tongue | 0 (0%) | 3 (8%) | 12 (30%) | 25 (63%) | 0 (0%) | 4 (10%) | 11 (28%) | 25 (63%) |
|  | Try to identify the glottis by inserting the blade as far as possible and slowly withdrawing it | 0 (0%) | 11 (28%) | 14 (35%) | 15 (38%) | 4 (10%) | 7 (18%) | 16 (40%) | 13 (33%) |
|  | Try to change the laryngoscope blade | 1 (3%) | 4 (10%) | 20 (50%) | 15 (38%) | 1 (3%) | 13 (33%) | 17 (43%) | 9 (23%) |
|  | Wait for an additional 30-60 seconds for the effect of rocuronium to take hold, or give a push | 2 (5%) | 6 (15%) | 13 (33%) | 19 (48%) | 4 (10%) | 12 (30%) | 12 (30%) | 12 (30%) |
|  | Try to remove the foreign body with a Magill forceps | 0 (0%) | 2 (5%) | 12 (30%) | 26 (65%) | 0 (0%) | 4 (10%) | 14 (35%) | 22 (55%) |
|  | Rotate the bougie to adjust the bevel of the endotracheal tube | 7 (18%) | 9 (23%) | 13 (33%) | 11 (28%) | 12 (30%) | 8 (20%) | 13 (33%) | 7 (18%) |
|  | Consider reducing the size of the endotracheal tube | 1 (3%) | 2 (5%) | 20 (50%) | 17 (43%) | 3 (8%) | 8 (20%) | 17 (43%) | 12 (30%) |
|  | Try changing the person performing the procedure | 1 (3%) | 4 (10%) | 9 (23%) | 26 (65%) | 10 (25%) | 8 (20%) | 13 (33%) | 9 (23%) |
|  | Consider using supraglottic devices | 5 (13%) | 9 (23%) | 11 (28%) | 15 (38%) | 16 (40%) | 10 (25%) | 8 (20%) | 6 (15%) |
|  | Consider performing surgical airway management | 0 (0%) | 0 (0%) | 9 (23%) | 31 (78%) | 0 (0%) | 8 (20%) | 10 (25%) | 22 (55%) |
|  | Consider reversing the anesthetic and returning to spontaneous breathing or BVM ventilation | 4 (10%) | 9 (23%) | 10 (25%) | 17 (43%) | 18 (45%) | 9 (23%) | 7 (18%) | 6 (15%) |
|  | Request support from a backup or senior physician | 1 (3%) | 4 (10%) | 9 (23%) | 26 (65%) | 9 (23%) | 9 (23%) | 8 (20%) | 14 (35%) |

Consensus (≥70% of ratings 3–4) was calculated from the distributions shown in this table.
