# Supplementary figures and images for: Implementation tendencies and expert perspectives on physician-performed prehospital endotracheal intubation in Japan: Findings from the first round of a Delphi survey
Source: PLoS One. 2026 Mar 30;21(3):e0346146. doi: 10.1371/journal.pone.0346146 (PMC13035153; doi:10.1371/journal.pone.0346146)

**Figure S1. Identification and Development Process of Delphi Survey Items.**


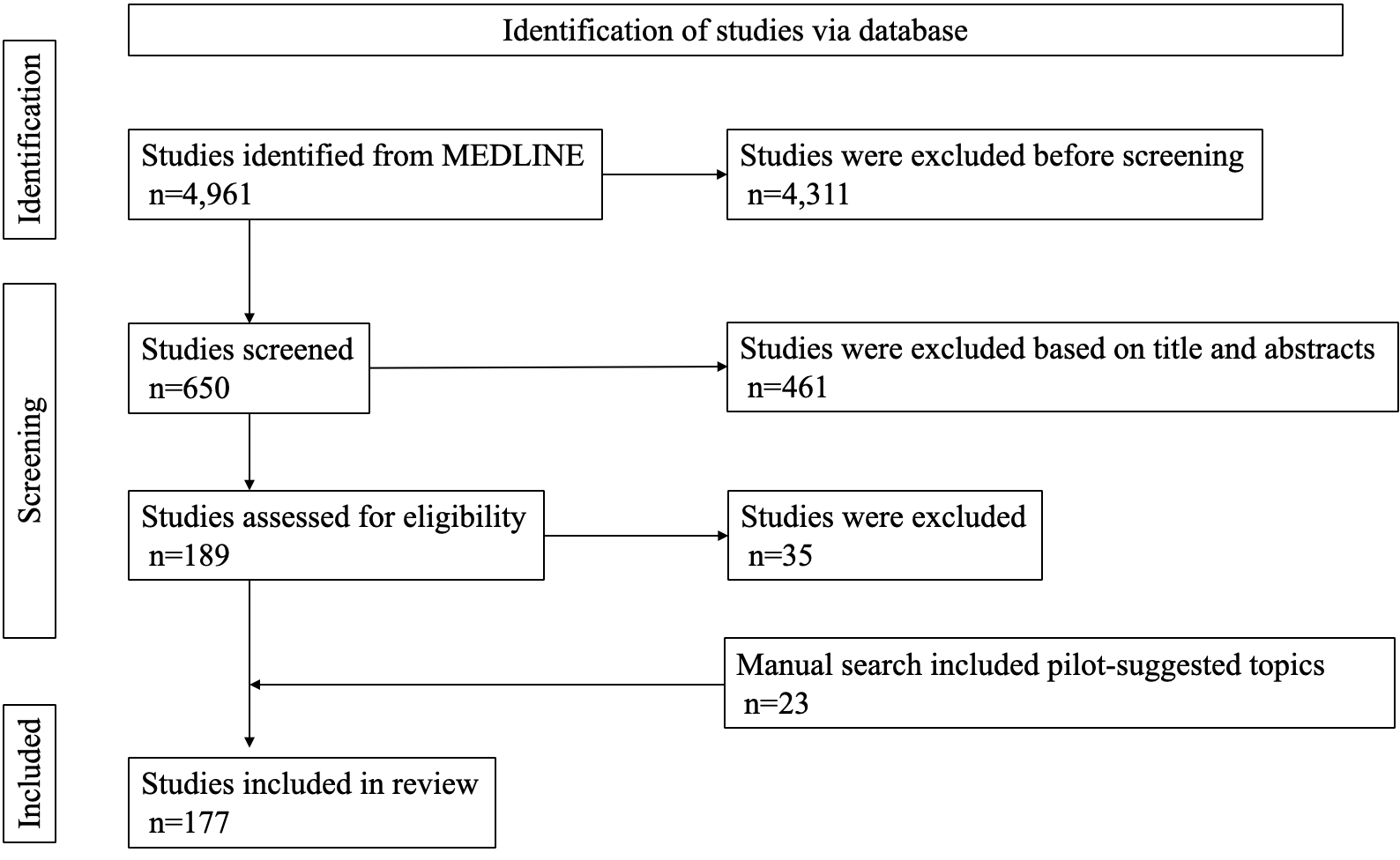

Supplement: S1 Fig — Flowchart illustrating the process of item generation for the Delphi survey, including literature review, pilot testing, and final categorization. Candidate items were identified through a literature review and a pilot study, followed by supplementary manual searches based on pilot feedback. The final set of items was organized chronologically and grouped into eight domains. (DOCX) [file pone.0346146.s003.docx]
