# Supplementary material for: Implementation tendencies and expert perspectives on physician-performed prehospital endotracheal intubation in Japan: Findings from the first round of a Delphi survey
Source: PLoS One. 2026 Mar 30;21(3):e0346146. doi: 10.1371/journal.pone.0346146 (PMC13035153; doi:10.1371/journal.pone.0346146)
Supplement: S1 File — Thematic analysis of contextual factors and suggestions raised by participants in open comment fields. (DOCX) [file pone.0346146.s004.docx]

**Text S1. Summary of Free-Text Comments from Round 1.**

1. **Environment during endotracheal intubation**

Some respondents noted that endotracheal intubation is occasionally performed at the rescue site prior to transferring the patient to the ambulance. Others commented that performing the procedure inside the ambulance is challenging due to limited 360-degree access. One participant mentioned that the meaning of “360-degree access” in the context of ambulance settings was unclear.

1. **Patients and indication**

Several respondents emphasized that physicians should be capable of performing endotracheal intubation independently during prehospital care. Others reported that, at their institutions, prehospital intubation is generally discouraged unless there are critical abnormalities in airway (A) or breathing (B) immediately prior to cardiac arrest.

1. **Operator Readiness and Role Assignments**

Some respondents expressed disagreement with the item suggesting the assignment of a backup intubator or ensuring that help can be contacted, noting that prehospital teams often operate with a single physician and that external assistance is not readily available. Communication via radio was mentioned as an alternative. Comments also noted that physicians undergoing on-the-job training (OJT) in helicopter emergency medical services may lack sufficient experience. In some systems, backup personnel are available for OJT physicians, but backup is typically not feasible when only one physician is dispatched.

1. **Oxygen and Management Equipment**

A range of views were expressed regarding equipment use. Some respondents reported routine use of the Macintosh laryngoscope, while others preferred video laryngoscopy for prehospital intubation. Several noted that bougies were not carried, and smaller endotracheal tubes were only prepared when intubation difficulty was anticipated. Additional comments included the limited availability of suction devices, rare use of gastric tubes in the prehospital setting, and infrequent reliance on mechanical ventilators—one respondent reported using a Jackson-Rees circuit after intubation. The use of qualitative CO₂ detectors and supraglottic airway devices was also described as uncommon.

1. **Peripheral equipment**

One respondent noted the availability of a transcutaneous thermometer but indicated that intubation was performed only after excluding hypothermia.

1. **Medications and Dosage Considerations**

In cases of anticipated hypotension, some respondents reported using norepinephrine or epinephrine (5 μg IV) as substitutes for phenylephrine. Midazolam was described as sufficient for pre-intubation sedation, with rocuronium administered when necessary. Several participants noted that ketamine, succinylcholine, and phenylephrine were not routinely stocked.

1. **Confirmation**

Some respondents indicated the use of ultrasound to assess the presence or absence of esophageal intubation and to detect lung sliding as confirmation of appropriate tube placement.

1. **Troubleshooting and Adjustments**

In cases of failed intubation, some respondents reported providing instructions to proceed with surgical airway management.
